# Support for Policies to Prohibit the Sale of Menthol Cigarettes and All Tobacco Products Among Adults, 2021

**DOI:** 10.5888/pcd20.220128

**Published:** 2023-02-02

**Authors:** Maeh Al-Shawaf, Kya N. Grooms, Margaret Mahoney, Natasha Buchanan Lunsford, Deirdre Lawrence Kittner

**Affiliations:** 1 Centers for Disease Control and Prevention, Office on Smoking and Health, Atlanta, Georgia; 2 Oak Ridge Institute for Science and Education, Oak Ridge, Tennessee

## Abstract

This study assessed support for commercial tobacco retail policies among adults. Data came from SpringStyles 2021, a web panel survey of adults in the US aged 18 years or older (N = 6,455). Overall, 62.3% of adults supported a policy prohibiting the sale of menthol cigarettes, and 57.3% supported a policy prohibiting the sale of all tobacco products. A majority of adults supported tobacco retail policies aimed at preventing initiation, promoting quitting, and reducing tobacco-related disparities. These findings can help inform federal, state, and local efforts to prohibit the sale of tobacco products, including menthol cigarettes.

**Summary**

**What is already known on this topic?**

The tobacco retail environment contributes to commercial tobacco use initiation, tobacco consumption, tobacco product–related disparities, and lower likelihood of successful quitting.

**What is added by this report?**

In 2021, most adults supported a policy prohibiting the sale of menthol cigarettes and a policy prohibiting the sale of all tobacco products. We assessed support for these policies after some communities had already adopted local menthol sale prohibitions — a public health intervention that addresses racial and ethnic inequities in health.

**What are the implications for public health practice?**

Understanding population group differences in support for these policies can inform public health action and inform efforts to prohibit the sale of tobacco products, including menthol cigarettes.

## Objective

Commercial tobacco products, including menthol cigarettes, are disproportionately marketed and advertised to certain population groups (eg, people who are Black, youth, people who identify as LGBTQ+ [lesbian, gay, bisexual, transgender, queer or questioning]), increasing the likelihood of use and risk for tobacco-related harms among people in these groups (1,2). This might help to explain why disparities in tobacco product use persist, despite considerable declines in US cigarette smoking (3). Additionally, disparities exist in who is covered by comprehensive policies that reduce tobacco product use (4). Public opinion can serve as a lever and influential factor in the acceleration of policy adoption (5). As such, we assessed support among adults for policies aimed at reducing tobacco product use and associated disparities.

## Methods

We used data from SpringStyles, a web panel survey of adults in the US aged 18 years or older. Porter Novelli conducts SpringStyles via Ipsos’s KnowledgePanel; panel members are randomly recruited by mail by using address-based probability sampling. During late March to mid-April 2021, 6,455 participants completed SpringStyles (response rate, 59.1%). Data were weighted to match the US Census Bureau’s American Community Survey (ACS) proportions for demographic variables, including sex, age, household income, race and ethnicity, household size, education, census region, and metro status. The study was exempt from human subjects review because it was a secondary analysis of de-identified data.

To assess support for tobacco retail policies among adults, respondents were asked 1) “To what extent would you support a policy to prohibit the sale of menthol cigarettes?” and 2) “To what extent would you support a policy to prohibit the sale of all tobacco products?” Response options were “strongly support,” “somewhat support,” “somewhat oppose,” and “strongly oppose.” Adults who responded strongly support or somewhat support were considered to support such a policy, and adults who responded strongly oppose or somewhat oppose were considered to oppose such a policy.

Weighted point estimates and 95% CIs were calculated overall and by sociodemographic characteristics (sex, age, race and ethnicity, education, annual household income, US region), current tobacco product use (cigarette use, menthol cigarette use, and noncigarette tobacco product use), and support or opposition toward tobacco retail policies. We performed *χ* ^2^ tests to assess differences between support for policies, sociodemographic characteristics, and current tobacco product use. Significance was set at *P* < .05, and analyses were conducted using SAS version 9.4 (SAS Institute Inc).

## Results

Demographic characteristics of respondents are shown in Table 1. Overall, 62.3% of adults supported a policy to prohibit the sale of menthol cigarettes (Table 2). Significant differences in support existed by sex (59.5% among men, 65.0% among women; *P* < *.*001); educational attainment (57.4% among adults with a high school diploma or less, 68.7% among adults with a college degree or higher; *P* < .001); annual household income (57.0% among adults with <$25,000 in annual household income, 64.4% among adults with ≥$75,000 in annual household income; *P = .*005); and by all 3 tobacco product-use variables (*P *< .001 for all). Approximately two-thirds of respondents who did not currently use tobacco products and more than one-third who currently used tobacco products, including menthol cigarettes, supported this policy. No differences in support for this policy were observed by age (*P* = *.*08), race and ethnicity (*P* = *.*44), or US region (*P* = *.*06) (Table 2).

**Table 1:** Demographic Characteristics of Participants, Study on Support for Policies to Prohibit the Sale of Menthol Cigarettes and the Sale of All Tobacco Products Among Adults in the US, SpringStyles, 2021

Characteristic	No.	Weighted % (95% CI)
**Overall**	6,455	—
**Sex**		
Male	3,014	48.4 (46.9–49.9)
Female	3,441	51.6 (50.1–53.1)
**Age, y**		
18−29	564	19.7 (18.2–21.2)
30−44	1,785	25.4 (24.1–26.6)
45−59	2,131	25.3 (24.1–26.4)
≥60	1,975	29.7 (28.4–31.0)
**Race and ethnicity**		
Hispanic	730	16.6 (15.3–17.8)
Non-Hispanic Black	517	11.8 (10.7–12.9)
Non-Hispanic other	295	6.9 (6.1–7.8)
Non-Hispanic White	4,710	64.7 (63.2–66.3)
**Educational attainment**		
High school or less	1,718	38.1 (36.5–39.6)
Some college	1,972	30.2 (28.8–33.0)
College degree or higher	2,765	31.8 (30.5–33.0)
**Annual household income, $**		
<25,000	604	12.3 (11.3–13.4)
25,000–49,999	962	17.5 (16.3–18.7)
50,000–74,999	1,121	17.3 (16.2–18.5)
≥75,000	3,768	52.8 (51.3–54.3)
**US Census region^a^**		
Northeast	1,133	17.3 (16.2–18.4)
Midwest	1,527	20.8 (19.7–22.0)
South	2,273	37.9 (36.5–39.4)
West	1,522	24.0 (22.7–25.3)
**Current cigarette use^b^**		
Yes	659	11.0 (10.0–11.9)
No	5,765	89.0 (88.1–90.0)
**Current menthol cigarette use^c^**		
Yes	352	6.4 (5.6–7.2)
No	5,736	93.6 (92.8–94.4)
**Current noncigarette tobacco use^d^**		
Yes	452	8.3 (7.4–9.2)
No	5,433	91.7 (90.8–92.6)
**Policy to prohibit the sale of menthol cigarettes^e^**		
Strongly support	2,018	31.2 (29.8–32.6)
Somewhat support	1,958	31.1 (29.7–32.5)
Somewhat oppose	1,275	19.9 (18.7–21.1)
Strongly oppose	1,056	17.7 (16.6–18.9)
**Policy to prohibit the sale of all tobacco products^f^**		
Strongly support	1,896	30.0 (28.7–31.4)
Somewhat support	1,706	27.3 (26.0–28.7)
Somewhat oppose	1,396	21.8 (20.5–23.0)
Strongly oppose	1,355	20.9 (19.7–22.1)

Abbreviation: — , not applicable.

a Northeast: Connecticut, Maine, Massachusetts, New Hampshire, New Jersey, New York, Pennsylvania, Rhode Island, and Vermont; Midwest: Illinois, Indiana, Iowa, Kansas, Michigan, Minnesota, Missouri, Nebraska, North Dakota, Ohio, South Dakota, and Wisconsin; South: Alabama, Arkansas, Delaware, District of Columbia, Florida, Georgia, Kentucky, Louisiana, Maryland, Mississippi, North Carolina, Oklahoma, South Carolina, Tennessee, Texas, Virginia, and West Virginia; West: Alaska, Arizona, California, Colorado, Hawaii, Idaho, Montana, Nevada, New Mexico, Oregon, Utah, Washington, and Wyoming.

b Current cigarette use was defined as respondents who reported cigarette use at least once during the past 30 days.

c Respondents who reported cigarette use at least once during the past 30 days were asked “When you smoke cigarettes, how often do you smoke menthol cigarettes?” Current menthol cigarette use was defined as participant responses that included “all of the time,” “most of the time,” “sometimes,” or “rarely.” Noncurrent menthol use was defined as participants who responded “never.”

d Respondents were asked about the current (past 30 days) use of the following noncigarette tobacco products: cigars, including large cigars, cigarillos, or little cigars that look like cigarettes; electronic cigarettes (e-cigarettes) or other electronic vaping products; heated tobacco products, also known as “heat-not-burn”; nicotine pouches, lozenges, tablets, or gum, not used as quit medications; smokeless tobacco (chewing tobacco, snuff, dip, snus, dissolvable tobacco); water pipes, also known as hookahs, filled with tobacco; or some other tobacco product (eg, pipes filled with tobacco).

e Participants were asked, “To what extent would you support a policy to prohibit the sale of menthol cigarettes?”

f Participants were asked, “To what extent would you support a policy to prohibit the sale of all tobacco products?”

**Table 2:** Sociodemographic Characteristics and Tobacco Product Use Status, Study on Support for Policies to Prohibit the Sale of Menthol Cigarettes and the Sale of All Tobacco Products Among Adults in the US, SpringStyles, 2021

Characteristic	No.	Weighted % (95% CI)					
		Policy to prohibit the sale of menthol cigarettes^a^		*P* value^b^	Policy to prohibit the sale of all tobacco products^c^		*P* value^b^
		Support	Oppose		Support	Oppose	
**Overall**	6,455	62.3 (60.9–63.8)	37.7 (36.2–39.1)	—	57.3 (55.9–58.8)	42.7 (41.2–44.1)	—
**Sex**							
Male	3,014	59.5 (57.3–61.7)	40.5 (38.3–42.7)	<.001	52.2 (50.0–54.4)	47.8 (45.6–50.0)	<.001
Female	3,441	65.0 (63.0–67.0)	35.0 (33.0–37.0)		62.2 (60.2–64.2)	37.8 (35.8–39.8)	
**Age, y**							
18−29	564	63.7 (59.4–68.1)	36.3 (31.9–40.6)	.08	61.7 (57.3–66.0)	38.3 (34.0–42.7)	.03
30−44	1,785	60.2 (57.3–63.0)	39.8 (37.0–42.7)		55.7 (52.8–58.6)	44.3 (41.4–47.2)	
45−59	2,131	60.6 (58.0–63.2)	39.4 (36.8–42.0)		55.1 (52.5–57.7)	44.9 (42.3–47.5)	
≥60	1,975	64.8 (62.5–67.1)	35.2 (32.9–37.5)		57.7 (55.4–60.1)	42.3 (39.9–44.6)	
**Race and ethnicity**							
Hispanic	730	63.6 (59.4–67.8)	36.4 (32.2–40.6)	.44	60.5 (56.2–64.8)	39.5 (35.2–43.8)	<.001
Non-Hispanic Black	517	61.5 (56.5–66.5)	38.5 (33.5–43.5)		63.4 (58.4–68.3)	36.6 (31.7–41.6)	
Non-Hispanic other	295	66.7 (60.3–73.0)	33.3 (27.0–39.7)		67.4 (61.1–73.8)	32.6 (26.2–38.9)	
Non-Hispanic White	4,710	61.6 (60.0–63.3)	38.4 (36.7–40.0)		54.4 (52.7–56.1)	45.6 (43.9–47.3)	
**Educational attainment**							
High school or less	1,718	57.4 (54.7–60.2)	42.6 (39.8–45.3)	<.001	56.3 (53.6–59.1)	43.7 (40.9–46.4)	.008
Some college	1,972	61.7 (59.1–64.2)	38.3 (35.8–40.9)		55.1 (52.5–57.8)	44.9 (42.2–47.5)	
College degree or higher	2,765	68.7 (66.7–70.8)	31.3 (29.2–33.3)		60.6 (58.5–62.8)	39.4 (37.2–41.5)	
**Annual household income, $**							
<25,000	604	57.0 (52.2–61.8)	43.0 (38.2–47.8)	.005	54.3 (49.5–59.1)	45.7 (40.9–50.5)	.54
25,000–49,999	962	59.0 (55.3–62.8)	41.0 (37.2–44.7)		57.4 (53.6–61.1)	42.6 (38.9–46.4)	
50,000–74,999	1,121	63.0 (59.5–66.5)	37.0 (33.5–40.5)		58.3 (54.8–61.8)	41.7 (38.2–45.2)	
≥75,000	3,768	64.4 (62.5–66.3)	35.6 (33.7–37.5)		57.7 (55.8–59.7)	42.3 (40.3–44.2)	
**US Census region^d^**							
Northeast	1,133	64.4 (61.0–67.7)	35.6 (32.3–39.0)	.06	61.1 (57.7–64.5)	38.9 (35.5–42.3)	.004
Midwest	1,527	63.3 (60.3–66.4)	36.7 (33.6–39.7)		57.9 (54.8–61.0)	42.1 (39.0–45.2)	
South	2,273	59.8 (57.3–62.3)	40.2 (37.7–42.7)		54.1 (51.6–56.6)	45.9 (43.4–48.4)	
West	1,522	64.1 (61.0–67.1)	35.9 (32.9–39.0)		59.3 (56.2–62.3)	40.7 (37.7–43.8)	
**Current cigarette use^e^**							
Yes	659	36.7 (32.1–41.2)	63.3 (58.8–67.9)	<.001	25.2 (21.1–29.3)	74.8 (70.7–78.9)	<.001
No	5,765	65.4 (63.9–66.9)	34.6 (33.1–36.1)		61.2 (59.7–62.7)	38.8 (37.3–40.3)	
**Current menthol cigarette use^f^**							
Yes	352	35.4 (29.0–41.7)	64.6 (58.3–71.0)	<.001	28.6 (22.6–34.6)	71.4 (65.4–77.4)	<.001
No	5,736	65.9 (64.3–67.4)	34.1 (32.6–35.7)		61.3 (59.7–62.8)	38.7 (37.2–40.3)	
**Current noncigarette tobacco use^g^**							
Yes	452	33.9 (28.5–39.2)	66.1 (60.8–71.5)	<.001	22.9 (18.1–27.8)	77.1 (72.2–81.9)	<.001
No	5,433	67.4 (65.9–69.0)	32.6 (31.0–34.1)		63.6 (62.0–65.2)	36.4 (34.8–38.0)	

Abbreviation: — , not applicable.

a Participants were asked, “To what extent would you support a policy to prohibit the sale of menthol cigarettes?” Support included “strongly support” and “somewhat support” responses; oppose included “strongly oppose” and “somewhat oppose” responses.

b *P* value determined from χ^2^ test.

c Participants were asked, “To what extent would you support a policy to prohibit the sale of all tobacco products?” Support included “strongly support” and “somewhat support” responses; oppose included “strongly oppose” and “somewhat oppose” responses.

d Northeast: Connecticut, Maine, Massachusetts, New Hampshire, New Jersey, New York, Pennsylvania, Rhode Island, and Vermont; Midwest: Illinois, Indiana, Iowa, Kansas, Michigan, Minnesota, Missouri, Nebraska, North Dakota, Ohio, South Dakota, and Wisconsin; South: Alabama, Arkansas, Delaware, District of Columbia, Florida, Georgia, Kentucky, Louisiana, Maryland, Mississippi, North Carolina, Oklahoma, South Carolina, Tennessee, Texas, Virginia, and West Virginia; West: Alaska, Arizona, California, Colorado, Hawaii, Idaho, Montana, Nevada, New Mexico, Oregon, Utah, Washington, and Wyoming.

e Current cigarette use was defined as respondents who reported cigarette use at least once during the past 30 days.

f Respondents who reported cigarette use at least once during the past 30 days were asked “When you smoke cigarettes, how often do you smoke menthol cigarettes?” Current menthol cigarette use was defined as participant responses that included “all of the time,” “most of the time,” “sometimes,” or “rarely.” Noncurrent menthol use is defined as participants who responded “never.”

g Respondents were asked about the current (past 30 day) use of the following noncigarette tobacco products: cigars, including large cigars, cigarillos, or little cigars that look like cigarettes; electronic cigarettes (e-cigarettes) or other electronic vaping products; heated tobacco products, also known as “heat-not-burn”; nicotine pouches, lozenges, tablets, or gum, not used as quit medications; smokeless tobacco (chewing tobacco, snuff, dip, snus, dissolvable tobacco); water pipes, also known as hookahs, filled with tobacco; or some other tobacco product (eg, pipes filled with tobacco).

In addition, 57.3% of respondents supported a policy to prohibit the sale of all tobacco products (Table 2). Significant differences in support were found by sex (52.2% among men, 62.2% among women; *P* < .001); age (57.7% of respondents aged ≥60 y, 61.7% of respondents aged 18–29 y; *P* = *.*03); race and ethnicity (54.4% among non-Hispanic White adults, 60.5% among Hispanic adults, 63.4% among non-Hispanic Black adults, and 67.4% among non-Hispanic adults from other racial and ethnic population groups; *P *< .001); educational attainment (56.3% among adults with a high school diploma or less, 60.6% among adults with a college degree or higher; *P* = *.*008); US region (54.1% among respondents in the South, 61.1% among respondents in the Northeast; *P* = *.*004); and by all 3 tobacco product-use variables (*P* < .001). More than 61% of respondents who did not currently use tobacco products and about one-fourth of respondents who currently used tobacco products supported this policy. No differences in policy support existed by annual household income (*P* = *.*54) (Table 2).

## Discussion

Nearly two-thirds of adults supported prohibiting menthol cigarette sales, while more than half of adults supported prohibiting all tobacco product sales. Support for these policies was similar, which may be explained by general support for tobacco retail policies. Policy support varied by certain demographic characteristics and current tobacco product use. Our findings are generally consistent with previous research showing support for menthol cigarette sales prohibitions, including among population groups historically targeted by unjust marketing practices and with a high prevalence of menthol cigarette use (eg, non-Hispanic Black adults) (6,7). No significant racial and ethnic group differences were found regarding support of a policy to prohibit menthol cigarette sales, highlighting broad support among all respondents. Although support for both policies was lower among people who reported current tobacco product use compared with those who did not, more than one-third of respondents who currently smoked cigarettes or menthol cigarettes still supported prohibiting menthol cigarette sales, and more than one-quarter of these respondents supported prohibiting all tobacco product sales. In April 2021, the US Food and Drug Administration announced plans to ban menthol cigarettes and all characterizing flavors in cigars (8). As of February 2022, at least 145 US communities prohibit menthol cigarettes and other flavored product sales, and 2 cities prohibit all tobacco product sales (9,10). Given variations in support and the potential of ongoing policy adoption, continued education can demonstrate how policies can reduce overall tobacco product use and related disparities.

Our study has limitations. SpringStyles is a web panel survey and is not representative of the US adult population. However, data were weighted to match the ACS proportions, and previous estimates are consistent with other cross-sectional national household surveys (11). Because data were self-reported, the possibility of underreporting of certain variables exists. Although some communities had already adopted local menthol sale prohibitions or prohibited all tobacco product sales, we could not assess whether respondents resided in areas covered by these prohibitions. Our study did not specify support for policies at a specific level of government (eg, local, state, federal). Finally, the survey did not include questions assessing demographic characteristics (eg, people who identify as LGBTQ+) of certain additional population groups that are disproportionately affected by tobacco product use.

Understanding population group differences in support for tobacco retail policies can inform public health education, surveillance, evaluation, and programs. Moreover, these findings can inform federal, state, and local efforts to prohibit all tobacco product sales, including menthol cigarettes, reduce tobacco use and tobacco-related disparities, and advance health equity.
